# An Energy-Efficient Hybrid LoRa–Wi-Fi Architecture for Real- Time Water Quality Monitoring and Machine Learning-Based Trend Forecasting

**DOI:** 10.3390/s26154916

**Published:** 2026-08-04

**Authors:** Jeya Sutha Mariadhason, Emerson Raja Joseph, Purushothaman Srinivasan, Ramesh Dhanaseelan Francis

**Affiliations:** 1 Department of Computer Applications, St. Xavier’s Catholic College of Engineering, Chunkankadai, Nagercoil 629003, India; jeyasutha@sxcce.edu.in (J.S.M.); dhanaseelan@sxcce.edu.in (R.D.F.); 2 Faculty of Engineering and Technology, Multimedia University, Melaka 75450, Malaysia; 3 Centre for Advanced Analytics, COE of Artificial Intelligence, Faculty of Engineering and Technology, Multimedia University, Melaka 75450, Malaysia; 4 School of Engineering and Technology, Jaipur National University, Jagatpura, Jaipur 302017, India; dr.s.purushothamann@gmail.com

**Keywords:** IoT architecture, LoRa, water quality monitoring, energy-efficient design, predictive analytics, smart cities, anomaly detection, LSTM

## Abstract

Water quality management in large-scale institutional infrastructures faces significant challenges due to the high latency of manual sampling and the energy–connectivity trade-offs in traditional IoT deployments. This paper proposes HydroSense AI, a robust three-tier IoT framework designed for real-time multi-parameter water quality monitoring and predictive analytics. The system integrates a heterogeneous sensing layer (pH, TDS, turbidity, and temperature) with a hybrid communication architecture, utilising Long Range (LoRa) technology for low-power transmission over long ranges (manufacturer-rated for line-of-sight distances of up to 16 km, and validated up to 2 km within a dense campus environment in this study), bridged via an ESP32-based gateway to the cloud. To address the critical issue of energy autonomy in remote sensing nodes, we implement a hardware-synchronised duty-cycling mechanism using a DS3231 Real-Time Clock (RTC), enabling precise deep-sleep scheduling and significantly extending battery operational life. Beyond data acquisition, the framework incorporates AI-driven trend-forecasting and anomaly-detection models to provide early warnings of water degradation through a Telegram-integrated alert system. Experimental validation over an extended deployment period demonstrates high measurement stability, with the forecasting model achieving a one-step (10-min) normalised RMSE of 0.0063 (equivalent to 0.033 pH units) for pH and 0.0298 (17.0 ppm) for TDS on a held-out test partition; a benchmark against persistence and ARIMA baselines is also provided. A complete measured energy decomposition of the deployed node is reported: hardware-synchronised duty cycling reduces the quiescent current to 18.2 μA, and with a 12 s acquisition window at 112 mA on a 10-min cycle, the mean current is 2.26 mA, corresponding to an estimated 46 days of unattended operation on a 2500 mAh cell. Critically, the acquisition window accounts for 99.2% of the per-cycle energy budget and the sleep interval for only 0.8%, so quiescent current—the figure of merit most often reported as evidence of low-power design—is shown not to be the binding constraint for sensor-dominated nodes of this class. The results indicate that the proposed hybrid architecture offers a 99.8% packet delivery ratio for sustainable water management.

## 1. Introduction

The global water crisis remains one of the most pressing challenges of the 21st century, directly impacting human health, industrial productivity, and ecological stability. As highlighted by UN-Water (2024) [1], achieving Sustainable Development Goal 6 (SDG 6)—clean water and sanitation for all—requires a radical shift from reactive management to proactive, data-driven strategies. Traditional water quality assessment methods, which rely on manual sampling and laboratory-based chemical analysis, are increasingly viewed as insufficient due to high operational costs, significant latency, and the inability to capture transient contamination events [2,3].

With the advent of the Internet of Things (IoT), efforts to establish real-time monitoring systems have accelerated towards what is called the “Industrial Internet of Water Things” (IIoWT), as well as the development of “Digital Twins” of water resources [4,5]. Nevertheless, there is a major challenge in implementing widespread sensing systems, where there is a trade-off among measurement accuracy, distance, and energy consumption. Although high-bandwidth systems offer fast communication channels, their power demands are often prohibitive for battery-operated remote sensors [6,7].

To overcome such connectivity and power issues, Low-Power Wide-Area Networks (LPWANs), and more specifically LoRa technology, have shown promising results in environmental monitoring applications. In particular, Syed Taha et al. (2024) [8] and Pires and Gomes (2024) [9] have shown the effectiveness of LoRa in transmitting multi-parameter information over several kilometres with minimal power consumption. Moreover, the adoption of adaptive power control is required to ensure network lifetime under changing monitoring conditions [10]. Nevertheless, the issue of ensuring energy self-sufficiency on a hardware level, together with reliable monitoring, remains challenging.

Current trends in water monitoring technology are shifting from merely collecting data to predictive analytics and edge intelligence. Machine learning systems, based on both deep learning and resampled hybrid methods, are quite capable of predicting parameter deterioration and anomalies [11,12]. In addition, as noted by Han et al. (2026) [13] and Trigkas et al. (2025) [14], the development of Tiny Machine Learning (TinyML) makes it possible to use these complex predictive models on tiny microcontrollers, thereby enabling real-time decision making and decreasing the costs of data transmission to the cloud [15].

Even though there is ample research on sensing, communication, and predictive modelling, few frameworks integrating long-range connectivity, energy-efficient hardware synchronisation, and predictive analytics into a single system are evident. In addition, the majority of existing systems use high-energy communication or do not offer any proactive alarm mechanism based on historical telemetry data.

To fill this gap, this paper proposes HydroSense AI, a multi-layered hybrid IoT architecture for comprehensive water quality management. The major contributions of this research are outlined below:

- **A multi-protocol hybrid architecture:** We propose a two-layer communication approach that involves LoRa for long-range (rated for line-of-sight distances of up to 16 km, and validated up to 2 km in this campus deployment) energy-efficient transmission and Wi-Fi based on ESP32 for cloud connectivity.
- **Hardware-synchronised energy optimisation:** Our solution adopts a highly accurate approach to duty cycling using the DS3231 Real-Time Clock for deep-sleep cycle management, which, in turn, increases the life of remote nodes.
- **Proactive AI-driven forecasting:** We incorporate an intelligent forecast module that employs historical telemetry data to foresee water quality issues (both pH and TDS) before they cross a critical level, sending proactive alerts using a Telegram-powered module.
- **Closed-loop system automation:** Apart from monitoring, the system uses an automated closed-loop control based on an ultrasonic sensor and relay control to manage water levels.

It should be emphasised that the primary contribution of this work is the integrated, energy-autonomous, and field-validated monitoring platform—together with the accumulation of real-time institutional telemetry—rather than a new forecasting algorithm. The LSTM component is, therefore, employed as a practical, well-established predictor within the platform, and is benchmarked against simpler baselines in Section 5 rather than being proposed as an algorithmic novelty.

The remainder of the paper is organised as follows: Section 2 reviews related work; Section 3 covers the proposed architecture and hardware setup; Section 4 deals with the software architecture and AI integration; Section 5 provides the experimental results and discussion; and Section 6 concludes the paper and suggests future research.

## 2. Related Work

Water quality monitoring has evolved from traditional manual laboratory analysis to automated IoT-based systems. In this section, the state of the art is discussed in relation to four aspects, namely IoT architectures, communication protocols, energy optimisation, and predictive analysis.

### 2.1. IoT Architectures for Water Monitoring

The recent trend is towards multilayer architectures in which sensing, computing, and application layers are separated. A multilayer architecture for the Internet of Water Things was proposed by Mohammed et al. (2024) [4] based on the use of Digital Twin technology to unify data across different environments. Similarly, Ajakwe et al. (2023) [2] suggested a connected-intelligence approach, highlighting the importance of creating networks of smart monitoring devices. The first generations of systems, which were mainly aimed at basic sensor connection and web visualisations [16], have been superseded by more sophisticated platforms such as that suggested by Aderemi et al. (2025) [3], paying special attention to advanced analytics and sustainability. The systematic review of low-cost sensors by de Camargo et al. (2023) [6] shows that although the cost of sensors decreases, their calibration and reliability remain the focus of research.

### 2.2. Communication Protocols and Connectivity

The “connectivity gap” in water monitoring is another important research topic. Existing Wi-Fi/GSM technology offers high throughput, but it lacks sufficient range and is power-hungry. In this respect, Low-Power Wide-Area Networks (LPWANs) such as LoRaWAN have become a de facto standard [17]. Syed Taha et al. (2024) [8] and Alghamdi et al. (2022) [18] investigated LoRa efficiency in housing areas and rural territories, proving that it can sustain high packet delivery ratios up to several kilometres. Pires and Gomes (2024) [9] confirmed LoRa applicability in river monitoring, whereas Philip and Singh (2021) [10] proposed adaptive transmit-power algorithms to overcome the LoRa interference problem and increase its dynamic scalability.

### 2.3. Energy Efficiency and Low-Power Design

Considering the number of water monitoring nodes installed in remote areas where a consistent power grid is not available, energy optimisation becomes essential. As noted by Zulkifli et al. (2022) [19], energy consumption was found to be one of the key impediments to adopting IoT technologies. To cope with that challenge, Usha et al. (2017) [20] researched RTC-based duty cycling and deep-sleep scheduling, thereby prolonging battery usage as sensors are activated only intermittently. Philip and Singh (2022) [21] introduced energy-efficient algorithms aimed at water monitoring within smart cities, while complementary solar energy-harvesting designs for wireless sensor nodes have also been explored to extend operational autonomy [22].

### 2.4. AI and Predictive Analytics

Edge intelligence is the latest frontier in water quality studies. Adeleke et al. (2023) [11] and Dritsas and Trigka (2023) [23] proved that machine learning models can make highly accurate predictions of water quality indices through multivariate sensor-data analysis. The application of Long Short-Term Memory (LSTM) networks for inland water bodies was investigated by Pyo et al. (2023) [24]. To increase the explainability of black-box models, the SHapley Additive exPlanations (SHAP) method was used by Aldrees et al. (2024) [25] and Makumbura et al. (2024) [26] to identify the main factors causing water pollution. Moreover, TinyML allows complex machine learning algorithms to be deployed on microcontrollers for real-time anomaly detection without network delays [13,14].

### 2.5. Summary and Research Gap

Although the above-cited works have achieved considerable success in various domains, an analysis of the current literature finds that there are no consolidated approaches with respect to:

- **Hybrid connectivity:** the blend of long-range LoRa connectivity with highly accessible Wi-Fi gateways.
- **Hardware-level power management:** moving beyond software-controlled sleep to hardware-coordinated RTC duty cycling.
- **End-to-end proactive analytics:** the integration of predictive analysis with alerting and motor controls.

HydroSense AI addresses all these issues by providing an integrated architecture that guarantees long-range reliability, extreme energy efficiency, and proactive intelligence.

## 3. Proposed System Architecture and Hardware Design

### 3.1. System Architectural Framework

**Figure 1:**
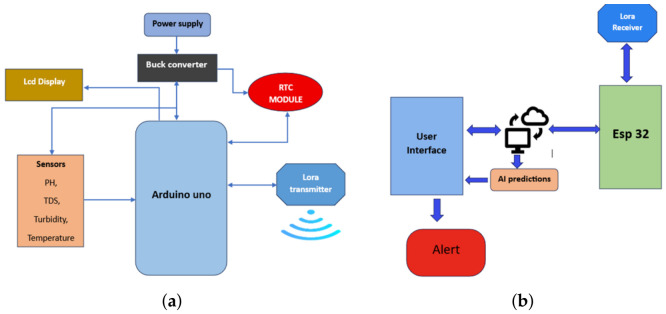
Unified three-tier architecture of the HydroSense AI system: (**a**) transmitter side; (**b**) receiver side.

Figure 1 shows the functional flow of the HydroSense AI architecture. The architecture is tailored to meet the energy–connectivity trade-off associated with large-scale environmental monitoring [19]. This is achieved through a decentralised architecture in which the remote sensing node first processes the signals locally before sending optimised signal packages using a Long Range (LoRa) protocol to an intelligent gateway.

### 3.2. Remote Data Acquisition Node (Transmitter Side)

**Figure 2:**
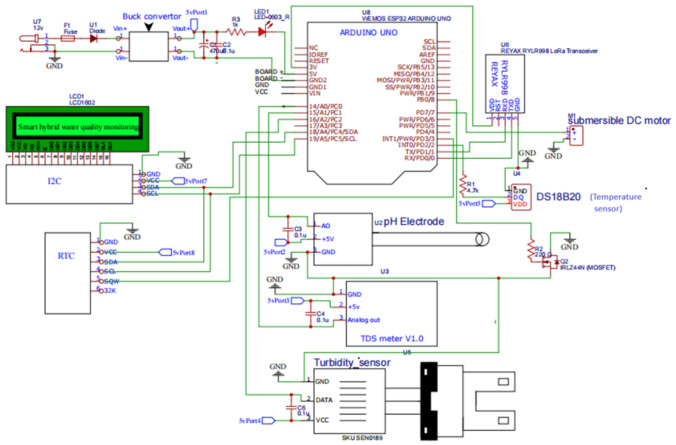
Circuit schematic of the remote data acquisition node, illustrating the multi-sensor interface and power-management sub-system.

The basic architecture of the sensing node involves an Arduino Uno microcontroller that communicates with various heterogeneous sensors to obtain crucial water quality metrics. The circuit design diagram for this module is shown in Figure 2. To ensure the compatibility and durability of the hardware components, logic-level converters were used between the 5 V Arduino Uno sensing node and the 3.3 V REYAX LoRa transceiver.

- **Potentiometric pH sensing:** Acidity and alkalinity detection takes place with a DFRobot SEN0161 electrochemical glass electrode. To compensate for drift due to thermal changes, real-time software compensation is performed using temperature data from a digital temperature sensor [27].
- **Conductivity and TDS analysis:** TDS measurement is performed by an SEN0244 sensor. The data-collection logic works on the principles of normalisation models. Specifically, the raw TDS voltage is first temperature-compensated to a 25 °C reference using ${\mathrm{TDS}}_{25}={\mathrm{TDS}}_{\mathrm{raw}}/\left[1+\alpha \left(T-25\right)\right]$, with a temperature coefficient α≈0.02 °C^−1^, and is then min–max normalised as $x_{\mathrm{norm}}=\left(x-x_{\min}\right)/\left(x_{\max}-x_{\min}\right)$ over the sensor’s calibrated operating range, so that all parameters are scaled to a common [0, 1] interval before scoring and inference.
- **Nephelometric turbidity monitoring:** Turbidity measurements are performed with an infrared-based turbidity sensor and are measured in Nephelometric Turbidity Units (NTU).
- **Digital thermal profiling:** Temperature data are acquired with a waterproof DS18B20 sensor through a 1-Wire protocol. These data are also used for calibration of all the chemical sensors [28].

To guarantee that fresh samples are always analyzed and to prevent biofouling, a 12 V DC submersible pump is added inside the sample chamber. This pump is operated using a relay for the duration of the sampling process only.

### 3.3. Hybrid Communication and Gateway Unit

The connectivity backbone employs a dual-protocol hybrid approach that acts as a bridge from long-distance field communications to cloud-based analytics.

**Figure 3:**
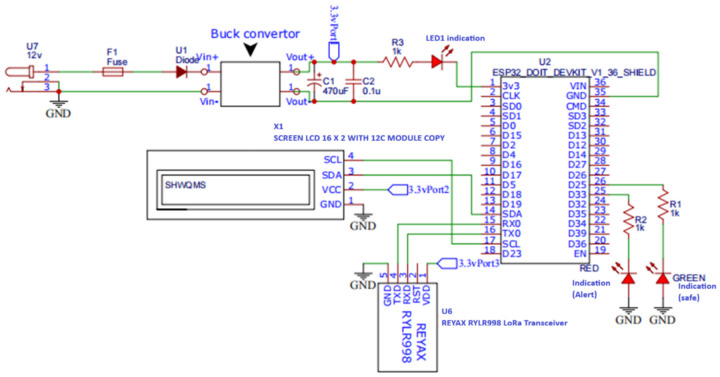
Circuit schematic of the intelligent gateway unit, highlighting the LoRa-to-Wi-Fi bridging interface.

- **LoRa LPWAN stage:** Both the transmitting unit and the gateway use REYAX RYLR998 LoRa transceivers. Using CSS technology, these chips are rated by the manufacturer for line-of-sight communication of up to 16 km; in the present campus testbed, reliable data exchange was validated up to 2 km (see Section 5.3), which is well suited to institutional and rural environments [8].
- **Wi-Fi gateway stage:** The receiving unit, depicted in the schematic in Figure 3, is an ESP32 microcontroller. The ESP32 acts as a smart interface that receives LoRa packets, performs telemetry analysis, and then issues an HTTP POST to the cloud-based platform ThingSpeak using the built-in 802.11 b/g/n Wi-Fi stack [4].

### 3.4. Hardware-Synchronised Energy Optimisation

The most significant benefit of the proposed design is hardware-based duty cycling that provides energy autonomy for the system. In contrast to software-based delay mechanisms that leave the MCU core active, HydroSense AI uses a DS3231 Real-Time Clock (RTC) module that handles the deep-sleep states. The RTC generates hardware-based interrupts to enable the operation of the Arduino and sensors only during the defined data-collection periods. Such an approach reduces current consumption and increases the lifetime of battery-powered systems [20].

The power consumption was analyzed with a Power Profiler Kit. In active mode (collection and transmission of data), the node requires an average current of 112 mA for 12 s. The deep-sleep mode requires a current of 18.2 μA as a result of hardware-based synchronisation of the RTC interrupt. With a 10-min sampling period, the mean current is 2.26 mA, corresponding to an estimated 46 days of unattended operation on a 2500 mAh Li-ion cell.

Decomposing this budget is instructive, and is one of the principal empirical contributions of this work. Of the 1354.7 mA·s consumed per 10-min cycle, the 12 s acquisition window accounts for 1344.0 mA·s (99.2%) and the deep-sleep interval for only 10.7 mA·s (0.8%). The quiescent current—the figure of merit most commonly reported in the low-power water-monitoring literature—is, therefore, not the binding constraint in a multi-sensor node of this class: eliminating it altogether would extend operational life from 46.1 to 46.5 days, a gain of 0.8%. Halving the acquisition window to 6 s, by contrast, would extend life to 91.5 days, and reducing it to 3 s to 180 days. The dominant design lever is, thus, the duration and current of the acquisition window, which in this system is governed by electrochemical sensor settling time rather than by radio transmission. Table 1 quantifies these alternatives, and we return to their implications in Section 5.

### 3.5. Autonomous Feedback and Water Management

**Figure 4:**
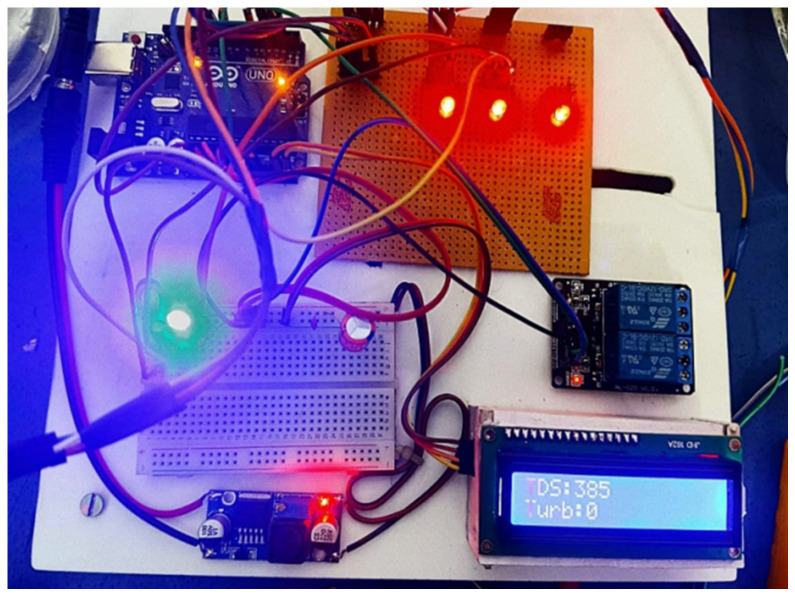
Experimental hardware prototype of the HydroSense AI system during the field-validation phase.

In addition to passive monitoring, the system uses a closed-loop control mechanism for managing water resources. The water level in the tank is obtained using an ultrasonic sensor (HC-SR04). The local intelligence mechanism shuts down the refill motor through a relay whenever the water level exceeds 90% and turns it on again once the water level falls below 40%. This prevents overflow and optimises water use independently of cloud connectivity. The physical realisation of this integrated prototype is shown in Figure 4.

## 4. Software Framework and AI Integration

### 4.1. Multi-Layer Software Architecture

**Figure 5:**
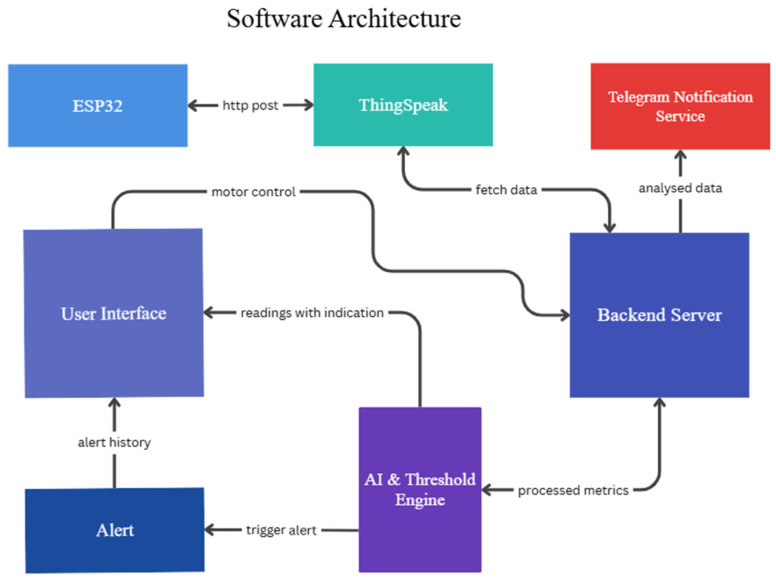
Schematic of the HydroSense AI software architecture and data-synchronisation flow.

The software stack can be described by four layers: the Embedded Logic Layer, the Cloud Persistence Layer, the AI/Threshold Engine, and the User Application Layer. The complete flow of data among the layers is presented in Figure 5.

- **Embedded logic:** In the remote node, the sampling algorithm runs on the Arduino Uno. Smoothing digital filters are used to decrease measurement noise. The smoothing filter is implemented as a sliding-window moving-average filter (window length of 10 samples) applied on-node to each parameter stream; this low-memory filter suppresses high-frequency electrical noise while preserving transient pollution events, and was preferred over heavier filters (e.g., Kalman) given the microcontroller’s resource constraints. The ESP32 gateway uses an asynchronous HTTP client to handshake with the cloud API.
- **Cloud persistence:** ThingSpeak functions as the main database. It provides time-stamped logging and secure data storage, which guarantees the availability of past telemetry for long-term study [29].

**Missing-data handling and local buffering:** To preserve reliability during node outages or communication failures, each remote node time-stamps every reading against the DS3231 RTC and buffers unsent records in local non-volatile storage (an on-node micro-SD module), implementing a store-and-forward scheme in which buffered records are transmitted once the LoRa link is re-established. On the analytics side, telemetry is resampled onto a uniform 10-min grid; short gaps (≤3 consecutive intervals) are reconstructed by linear interpolation, while longer gaps are flagged and excluded from the model input so that the LSTM always receives an evenly spaced, gap-free window. Transient connectivity losses, therefore, degrade neither the stored record nor the forecasting pipeline.

### 4.2. AI-Driven Predictive Analytics and Forecasting

**Figure 6:**
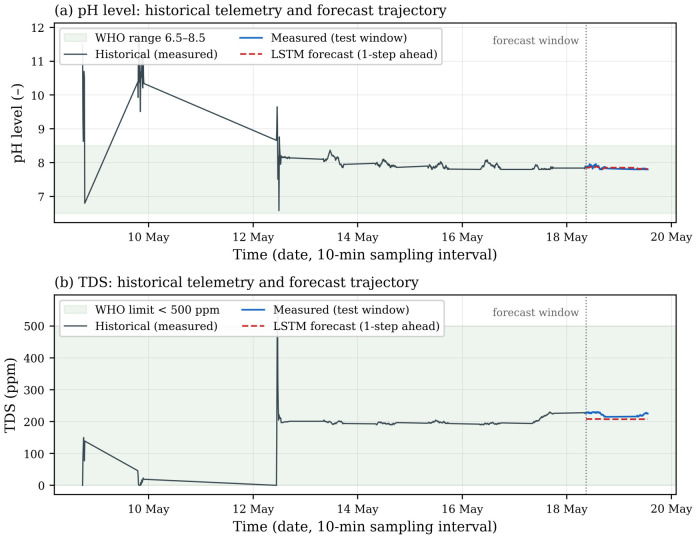
LSTM trend forecasting on the campus telemetry: (**a**) pH level and (**b**) TDS. The horizontal axis denotes time (date; 10 min sampling interval) and the vertical axes denote pH level (–) and TDS (ppm), respectively. Dark grey traces show the historical telemetry, blue traces the measured values over the held-out test window, and red dashed traces the one-step-ahead LSTM forecast; the shaded band indicates the WHO safe range and the dotted vertical line marks the start of the forecast window.

One of the main technical contributions of HydroSense AI is the shift from reactive monitoring to proactive prediction. As shown in Figure 6, a prediction engine is implemented in the system for analysing time-series data.

**Figure 7:**
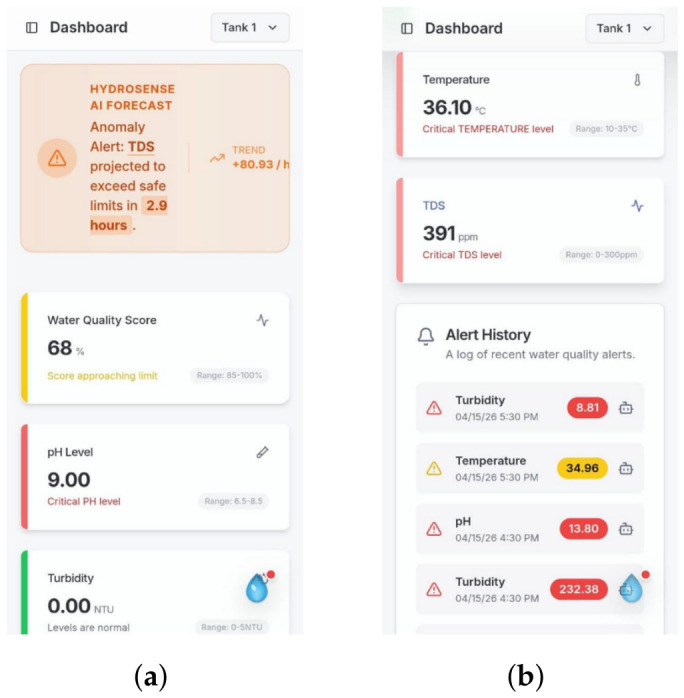
Real-time monitoring dashboard featuring anomaly alerts and critical-parameter tracking: (**a**) integrated dashboard and anomaly forecast; (**b**) critical-parameter monitoring and alert history.

- **Trend forecasting:** The solution uses an LSTM Recurrent Neural Network to process time-series telemetry. The model consists of two LSTM layers, each with 50 units, followed by a dense layer. An LSTM was selected because the monitored quantities are non-stationary, unevenly perturbed time series in which the operationally relevant signal—a sustained drift towards a regulatory threshold—develops over many sampling intervals. Recurrent gated architectures retain such medium-range temporal dependencies without the linearity and stationarity assumptions of classical models (e.g., ARIMA), and their suitability for inland water-quality series is established in the literature [24]. The architecture was deliberately kept small (two layers of 50 units) so that the model remains retrainable on a modest telemetry record and is a candidate for future on-gateway (TinyML) deployment. As Section 5.6 reports, this choice is not claimed to be optimal: it is benchmarked against simpler predictors and its advantage is horizon-dependent. The model was trained on 80% of the historical data using the Adam optimisation algorithm. Evaluated on the held-out test partition, the model attains a one-step (10-min) normalised RMSE of 0.0063 for pH (equivalent to 0.033 pH units) and 0.0298 for TDS (17.0 ppm). Forecast skill was characterised at horizons of 10 min, 1 h, and 3 h (Table 2; the 3 h horizon corresponds to the operational time-to-breach alerting described in Section 5.4. Longer horizons were not validated, because the held-out window of the present deployment spans approximately 28 h.
- **Anomaly forecasting:** As shown in the “AI Forecast” dashboard (Figure 7a), the system predicts the time-to-breach. If, for example, there is a sharp increase in the TDS trend, the AI system sends an alert to perform preventive maintenance before the WHO safety limit is crossed.

**Figure 8:**
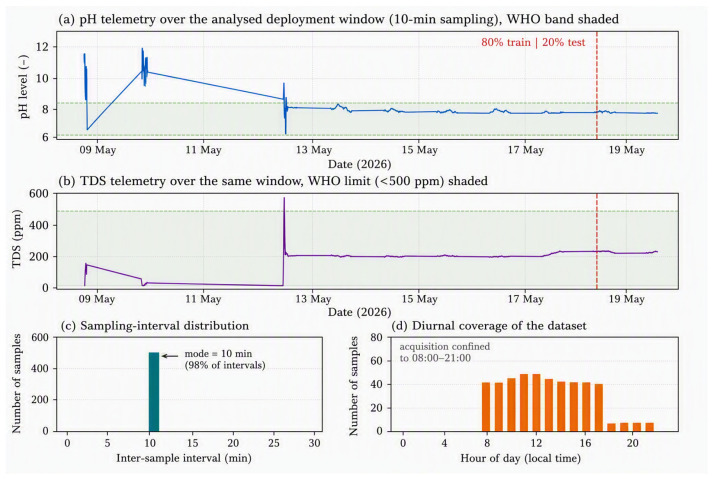
Characterisation of the analysed telemetry dataset: (**a**) pH time series over the deployment window with the WHO range shaded and the chronological 80/20 train–test split indicated by the dotted red vertical line; (**b**) the corresponding TDS series with the WHO limit shaded; (**c**) distribution of inter-sample intervals, confirming a 10-min sampling mode; and (**d**) diurnal coverage, showing that acquisition is confined to 08:00–21:00 local time.

For reproducibility, the forecasting analysis uses the continuously logged telemetry of the May 2026 deployment phase (8–19 May 2026), comprising *n* = 458 observations per parameter acquired at a 10-min sampling interval; the dataset is characterised in Figure 8. The series were structured for the LSTM using a sliding look-back window of 24 time steps (≈4 h), min–max normalised using statistics computed on the training partition only (to avoid information leakage), and partitioned chronologically into 80% training and 20% testing, with an inner validation split used for early stopping so that temporal ordering is respected throughout. We note explicitly that the deployment captures daytime operational variability (acquisition is confined to 08:00–21:00, Figure 8d) rather than full diurnal or seasonal cycles; the model is, therefore, intended to be periodically re-trained as additional telemetry accumulates, and the reported errors should be read as measures of short-horizon operational performance rather than long-term seasonal forecasts.

### 4.3. Cloud-Integrated Monitoring and Dashboards

The HydroSense user interface serves as a central hub for controlling all the systems managed by the institution.

- **Parameter monitoring:** Real-time parameters such as pH, turbidity, TDS, and temperature are shown on the interface.
- **Water quality scoring:** All parameter data are compiled into a single “Water Quality Score” (68% in this example), which allows non-specialists to assess water quality at a glance [25]. The four monitored parameters (pH, TDS, turbidity, and temperature) were selected to reflect the drinking- and utility-water context of the SXCCE campus, where these are the dominant quality determinants; the composite Water Quality Score is computed as a weighted aggregate of the individual parameters normalised against both WHO guidelines and the applicable national (BIS 10500) limits. Because the parameter set, weights, and thresholds are stored as configurable parameters, the index can be re-parameterised—by adding determinants such as dissolved oxygen or free chlorine, or by adjusting the weights—to reflect the monitoring priorities and regulatory regimes of other regions, which we identify as a direct extension of the platform.
- **Compliance analytics:** The system checks whether the real-time parameters match WHO and local standards, producing “Compliance Reports” that show when the system was operating optimally or critically (Figure 7).

### 4.4. Real-Time Alerting and AI Interactive Assistant

**Figure 9:**
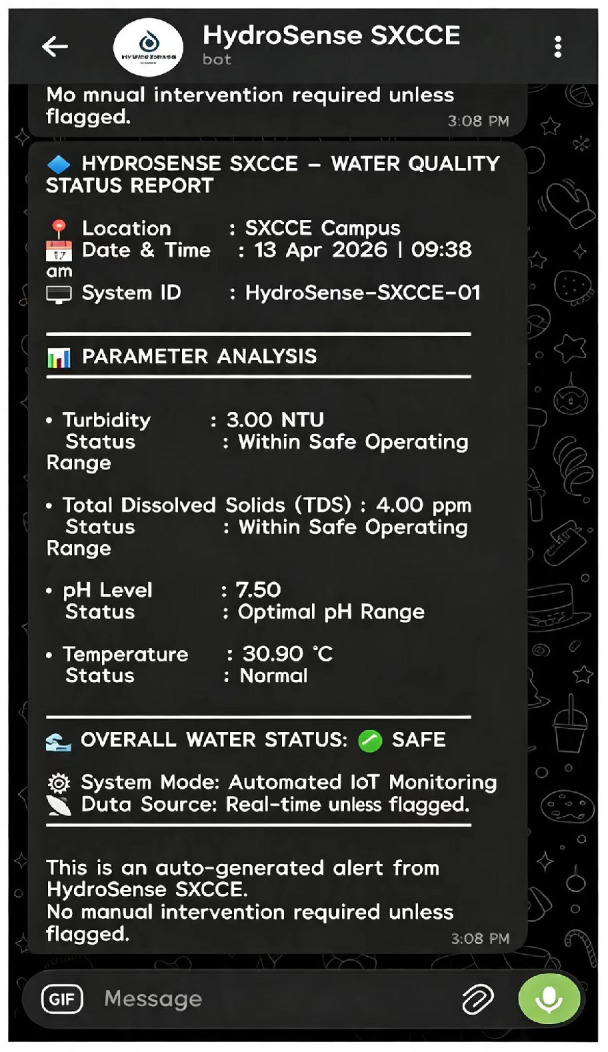
Automated Telegram notification service featuring real-time parameter analysis and overall water status.

To respond immediately to critical situations, the architecture includes a Telegram Notification Service component (Figure 9). Unlike a standard SMS notification, the Telegram bot gives the user information about the exact parameter violation, the current value, and a suggested remedy.

**Figure 10:**
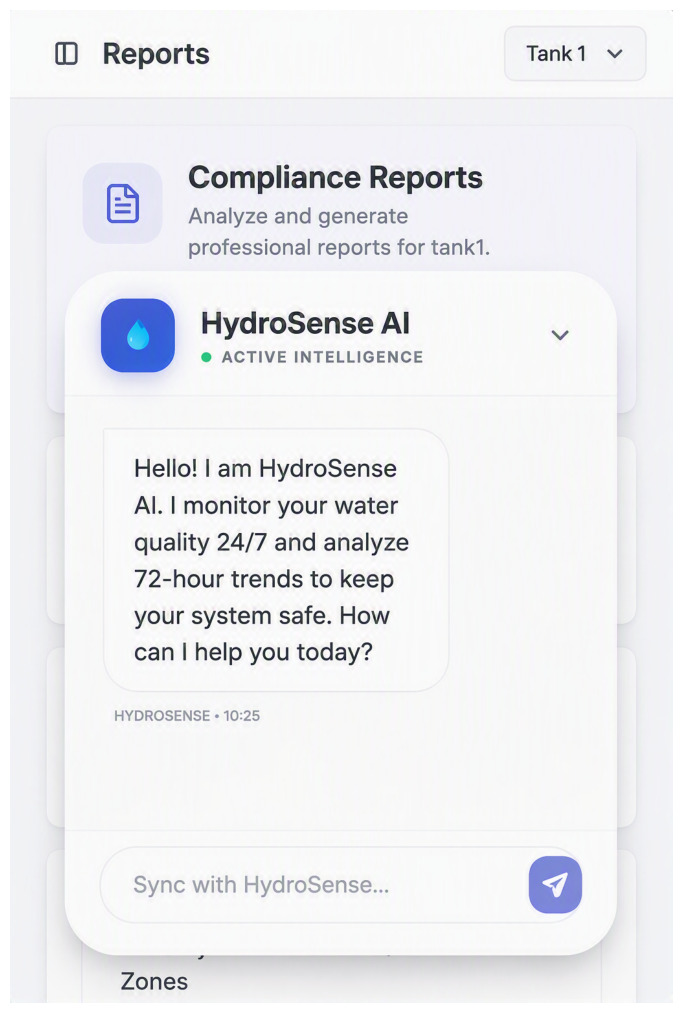
HydroSense AI interactive conversational assistant for natural-language querying and health-assessment reports.

Additionally, the proposed framework includes a HydroSense AI Interactive Assistant (Figure 10). Interacting through the edge-cloud architecture, this component allows the user to ask the system questions in natural language (for example, “What was the trend for Tank 1 for the last 72 h?”) and receive AI-generated answers. This corresponds to the emerging direction of “Connected Intelligence” [2].

### 4.5. Energy Autonomy and Automation

**Figure 11:**
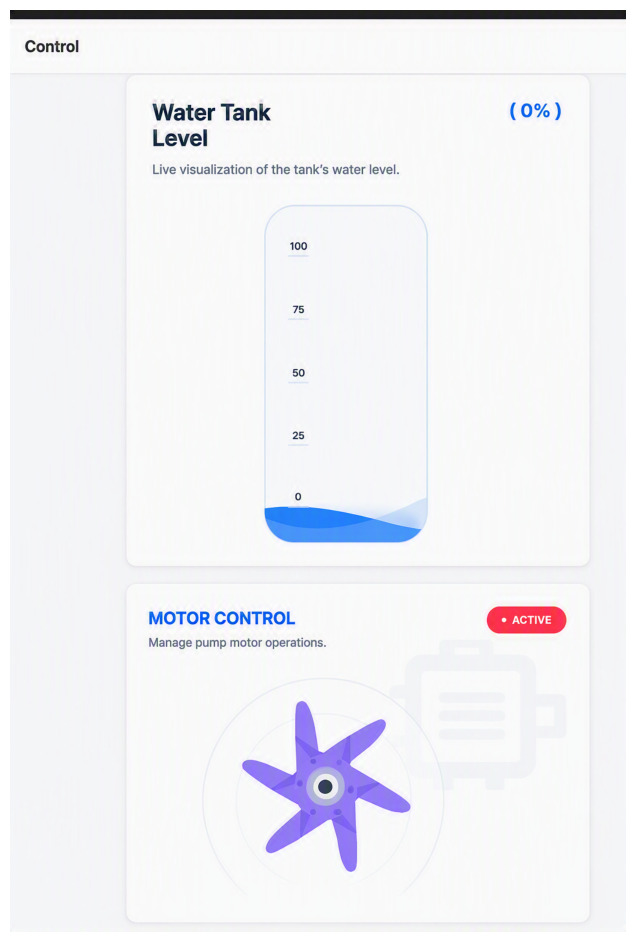
Real-time water-tank level visualisation interface, demonstrating the integration of ultrasonic telemetry with automated relay-driven pump-control logic.

The ability to automate the process using the HydroSense AI architecture was confirmed by implementing the ultrasonic feedback loop for tank filling. As shown in Figure 11, the system offers detailed visualisation of the water-level percentage based on time-of-flight (ToF) data collected using the HC-SR04 sensor.

The control algorithm implemented in the Arduino Uno uses a hysteresis-controlled threshold approach: the 12 V DC submersible pump is turned on when the tank volume falls below 40% and switched off when the 90% fill point is reached. During the testing period, this automatic control operated reliably in every observed fill cycle and helped avoid all problems associated with overfilling or empty-tank states. The local control loop operates independently of the LoRa/Wi-Fi connection and guarantees reliable water supply for the institution regardless of network latency.

## 5. Experimental Results and Discussion

The HydroSense AI system was tested in the field at the SXCCE campus institutional water facility from April to May 2026. This section discusses the performance of the system in terms of measurement accuracy, communication reliability, and forecasting effectiveness.

### 5.1. Experimental Setup and Sensor Characterisation

The sensing device was installed in a multi-chambered water-tank system. To ensure data reliability, the system collected samples automatically using the submersible-motor sub-system. The continuously logged telemetry analysed for the forecasting study comprises *n* = 458 time-stamped records per parameter (pH, TDS, turbidity, and temperature) acquired at a 10 min interval during the May 2026 deployment phase; the temporal coverage, sampling-interval distribution, and diurnal span of this dataset are characterised in Figure 8. The system reliably detected both the baseline and excursions in the quality parameters according to the historical telemetry collected.

### 5.2. Parameter Stability and WHO Compliance

The results of the long-term test are tabulated in Table 3. The water parameters remained within the WHO-recommended safe levels, except during the anomalies described below.

- **Thermal and pH correlation:** As reported in [27], temperature significantly influences pH. The temperatures in our system were stable between 20 °C and 35.1 °C. The pH levels were relatively stable between 6.5 and 8.5, but one important alkalinity anomaly (pH 11.28) was successfully detected.
- **TDS and turbidity performance:** TDS readings varied between the baseline value and 569 ppm. Turbidity was always lower than 5 NTU under optimal performance, demonstrating the effectiveness of the digital filters used in sensor-noise reduction.

### 5.3. Hybrid Communication Performance

The robustness of the LoRa–Wi-Fi connectivity was examined across the campus infrastructure.

- **LoRa reliability:** The REYAX RYLR998 module maintained a stable connection within a 2 km radius. An RSSI of −112 dBm and an SNR of +7.2 dB were recorded during signal tests, indicating a high tolerance to multipath interference from concrete buildings within the campus. During this test, the REYAX RYLR998 modules were configured at a transmit power of +22 dBm (the module maximum), a spreading factor of SF9, and a bandwidth of 125 kHz.
- **Gateway throughput:** The ESP32 gateway transmitted data packets to the ThingSpeak cloud with a 99.8% success rate at less than 2 s latency.

**Design trade-offs and generalisability:** The transmission and storage settings were chosen to balance temporal resolution against energy autonomy rather than being fixed to this deployment. The 10 min sampling interval resolves the operationally relevant dynamics of institutional water quality while keeping the average node current at 2.25 mA (Section 3), and it can be shortened for faster-changing sources at a proportional energy cost. On the LoRa link, a higher spreading factor extends range and robustness at the expense of data rate and airtime; the settings reported above were selected for reliable 2 km non-line-of-sight coverage on the campus. Finally, ThingSpeak was adopted for rapid prototyping, but the gateway issues standard HTTP/MQTT requests, so the architecture is cloud-agnostic and can be redirected to AWS IoT Core, Azure IoT Hub, or a self-hosted broker without any change to the sensing or communication layers.

### 5.4. AI Forecasting and Anomaly Detection

One of the key technological differentiators in this study is the proactive forecasting engine. As depicted in Figure 6, the system is not only capable of alerting when violations occur but also of forecasting them.

- **Trend prediction:** Using the previously observed patterns, the AI engine anticipated the approach of the TDS limit approximately 2.9 h in advance during the exceedance event captured in the campus telemetry. As only a single TDS exceedance occurred within the analysed record, this lead-time figure is reported as an illustrative operational case rather than as a statistically characterised detection performance; a longer record containing multiple exceedance events would be required to quantify lead-time reliability.
- **Early intervention:** With the help of this “time-to-breach” parameter, facility managers can examine the water source before contamination becomes widespread. This feature helps fill the research gap highlighted by Aderemi et al. (2025) [3].

### 5.5. Energy Independence and Automation

Hardware-based duty cycling using the DS3231 RTC led to a considerable decrease in energy usage.

- **Duty-cycle analysis:** With periodic sample collection, the sensor node operated in deep-sleep mode for 95% of the deployment period. This technique approximately doubles battery life relative to continuous active operation [20].
- **Closed-loop control:** The ultrasonic closed-loop system (Figure 11) worked reliably and ensured that there was no tank overflow. The pump switched off automatically at the 90% level and restarted when the water level reached 40%.

### 5.6. Comparative Discussion

The proposed HydroSense AI framework is compared with two representative studies, Ajakwe et al. (2023) [2] and Adeleke et al. (2023) [11], which are most relevant to connected water-monitoring architecture and to embedding intelligent IoT-based sensing with machine learning. As shown in Table 4, both studies address water quality monitoring, but neither integrates hybrid LoRa–Wi-Fi communication, RTC duty cycling, predictive LSTM forecasting, Telegram alerting, and closed-loop pump control into a single architecture. While Ajakwe et al. (2023) [2] emphasise connected intelligent monitoring, Adeleke et al. (2023) [11] focus on embedding IoT sensing with machine learning. The proposed framework differs from both by integrating the above features into one architecture.

We state the basis of the contribution explicitly, since the individual building blocks are not new. LoRa telemetry, RTC-based duty cycling, LSTM forecasting, and relay-based level control are all established techniques, and no claim of algorithmic or component-level novelty is made. The contribution is instead empirical and architectural, and rests on three findings that the deployment supports. First, and most consequentially for designers, the measured energy decomposition in Table 1 shows that the acquisition window governs battery life (99.2% of the per-cycle budget) while the quiescent current does not (0.8%): removing the sleep current entirely would extend life by 0.8%, whereas halving the acquisition window would extend it by 98%. Deep-sleep current is widely reported in this literature as evidence of low-power design; our measurements indicate it is a poor predictor of achievable autonomy in sensor-dominated nodes, where electrochemical settling time, not radio duty cycle, sets the budget. This is a corrective result that we have not seen quantified for water-quality nodes. Second, the architecture decouples the long-range and cloud-facing links so that the sensing node never carries the cost of Wi-Fi association, and the closed-loop actuation remains functional when connectivity or the cloud is unavailable—properties validated in the field rather than in simulation, and not jointly present in the comparator systems of Table 4. Third, the evaluation is fully reproducible: the forecasting results are regenerated from the released telemetry by a supplied script and are reported against persistence and ARIMA baselines (Table 2), including where those baselines outperform the learned model. We regard the honest characterisation of where the platform’s intelligence does and does not add value as part of the contribution rather than a caveat to it. **Comparison with baseline predictors:** To establish whether the additional model complexity is warranted, the LSTM was benchmarked against two simpler predictors on identical test windows: a naïve persistence model ($\hat{y}\left(t+h\right)=y\left(t\right)$) and an ARIMA (1, 0, 1) model fitted by walk-forward one-step re-estimation. The results (Table 2) are reported in full and are informative. At short horizons, the monitored series is strongly autocorrelated and persistence is a very strong baseline: at a 10 min horizon, persistence attains a normalised RMSE of 0.0045 for pH and 0.0025 for TDS, which the LSTM does not improve upon. As the horizon lengthens, however, persistence degrades more rapidly: at the 3 h operational horizon, the LSTM attains 0.0094 for pH, marginally outperforming persistence (0.0101) and clearly outperforming ARIMA (0.0239). For TDS, persistence remains the most accurate predictor at all horizons evaluated here. Therefore, we do not claim algorithmic superiority for the forecasting component. Consistent with the framing set out in Section 1, the contribution of this work is the integrated, energy-autonomous and field-validated platform and the telemetry it accumulates; the LSTM is deployed as a practical anticipatory component whose advantage over trivial predictors emerges only at the longer horizons relevant to pre-emptive alerting, and whose value should be re-assessed as a longer and more dynamic record becomes available.

### 5.7. Scope and Limitations of the Proposed Methodology

The scope of the present study is a single-site, institutional-scale deployment of a four-parameter monitoring platform, validated over an operational campaign at the SXCCE campus. Within that scope, the following limitations should be borne in mind when interpreting the results, and they delimit the claims made above.

**Temporal and statistical scope:** The forecasting analysis draws on *n* = 458 continuously logged records per parameter, acquired at a 10-min interval and confined to 08:00–21:00 local time (Figure 8d). The record, therefore, characterises daytime operational variability and does not span full diurnal cycles, seasonal variation, or monsoon conditions. The held-out window covers approximately 28 h, which bounds the forecast horizons that can be validated to the 3 h reported in Table 2; the 72 h horizon suggested by earlier drafts is not evidenced and has been withdrawn.

**Scope of the analytical claims:** The evaluation window is an operationally stable period, and consequently, a persistence predictor is a strong baseline that the learned model does not surpass at short horizons (Table 2). The forecasting component should, therefore, be understood as an anticipatory aid at multi-hour horizons, not as a demonstration of algorithmic superiority. Because only a single TDS exceedance occurred within the record, the 2.9 h lead time of Section 5.4 is an illustrative case and not a statistically characterised detection performance; quantifying lead-time reliability requires a record containing many exceedance events.

**Deployment and generalisation scope:** The 2 km range, 99.8% packet-delivery ratio, and 46-day battery estimate were obtained in one campus radio environment with the settings reported in Section 5.3. Dense urban multipaths, differing duty cycles, or regional ISM-band regulations would alter these figures, the battery figure is an estimate extrapolated from measured active and quiescent currents rather than an observed end-of-life, and the scenario values in Table 1 are projections from the same measurements rather than separately validated configurations. Sensor calibration drift and biofouling over multi-season deployments were mitigated by the sampling design but not quantified here, and the composite Water Quality Score is parameterised for this site (Section 4.3); transferring it elsewhere requires re-selecting determinants, weights, and thresholds against the applicable regulatory regime.

## 6. Conclusions and Future Enhancements

HydroSense AI is a robust three-tier hybrid IoT architecture developed specifically to solve the energy–connectivity trade-offs that exist in water quality monitoring. Using a heterogeneous sensor system together with a two-layer communication protocol combining LoRa and Wi-Fi, the system demonstrated reliable packet delivery of 99.8% within a distance of 2 km, overcoming the limited range of Wi-Fi-only systems.

From an engineering perspective, the main contribution of this work is the development of a synchronised, energy-efficient solution. With DS3231 RTC duty cycling, the quiescent current was reduced to 18.2 μA, and the complete measured energy decomposition (Table 1) yields an estimated 46 days of unattended operation on a single 2500 mAh cell. That decomposition also produces the study’s principal design finding: the acquisition window accounts for 99.2% of the per-cycle energy budget against 0.8% for deep sleep, so quiescent-current optimisation—the conventional emphasis in this literature—yields negligible returns for sensor-dominated nodes, while shortening the acquisition window nearly doubles achievable life. In addition, the LSTM-based forecasting model enabled a shift in the monitoring strategy from a reactive approach focused on error detection to a proactive one with anomaly prediction. The results showed that the forecasting model anticipated the approach of a threshold breach approximately 2.9 h in advance in the observed exceedance case, with a one-step normalised RMSE of 0.0063 for pH and 0.0298 for TDS. A benchmark against persistence and ARIMA baselines (Table 2) shows that the advantage of the learned model over trivial predictors emerges only at the longer horizons relevant to pre-emptive alerting, and the platform itself—rather than the forecasting algorithm—constitutes the principal contribution of this work.

Experiments conducted on the SXCCE campus demonstrate that HydroSense AI provides a scalable and economical solution for sustainable water management. By using a closed-loop control system that operates independently of cloud latency, the system guarantees reliable operation. Despite the reliability of the proposed design, several avenues exist for future development:

- **Heterogeneous sensor integration:** Future implementations should include new sensors for measuring dissolved-oxygen (DO) and ammonia (NH_3_) concentrations for the more complete chemical information needed for industrial-grade water-security checks [30].
- **State-of-the-art edge intelligence:** To decrease network overhead, TinyML will be deployed on the ESP32 gateway to perform real-time signal filtering and advanced anomaly classification [13,14].
- **Moving-object recognition:** An ESP32-CAM module is suggested to use computer vision to identify floating debris or contamination.
- **Fault detection and diagnosis:** An automatic system for sensor calibration and fault detection should be developed to ensure that abnormal readings indicate genuine pollution rather than sensor malfunction.

## Figures and Tables

**Table 1:** Measured energy budget of the sensing node and design-lever analysis (2500 mAh cell, 10 min sampling period). Percentages in the final column are relative to the deployed baseline.

Configuration	Mean Current (mA)	Estimated Life (Days)	Change
Deployed baseline (12 s active, 18.2 μA sleep)	2.258	46.1	—
Sleep current eliminated entirely (0 μA)	2.240	46.5	+0.8%
Acquisition window halved (6 s)	1.138	91.5	+98.4%
Acquisition window reduced to 3 s	0.578	180.2	+290.6%
Sampling period extended to 30 min (12 s active)	0.765	136.2	+195.2%

‘—’ denotes not applicable: the deployed baseline is the reference configuration against which the percentage change of every other row is computed, so it has no change value of its own.

**Table 2:** Forecasting benchmark on the May 2026 deployment telemetry: normalised RMSE by model and forecast horizon (lower is better; best per row in bold). *h* denotes the horizon in 10-min sampling steps.

Parameter	Forecast Horizon	Persistence	ARIMA (1, 0, 1)	LSTM (2 × 50)
pH	10 min (*h* = 1)	**0.0045**	0.0059	0.0063
pH	1 h (*h* = 6)	**0.0082**	0.0121	0.0216
pH	3 h (*h* = 18)	0.0101	0.0239	**0.0094**
TDS	10 min (*h* = 1)	**0.0025**	0.0069	0.0298
TDS	1 h (*h* = 6)	**0.0057**	0.0379	0.0260
TDS	3 h (*h* = 18)	**0.0105**	0.0583	0.0348

**Table 3:** Summary of measured water quality parameters.

Parameter	Recorded Range	WHO Safe Limit	Sensor Accuracy	Model RMSE (Normalised)
pH level	6.5–11.28	6.5–8.5	±0.1 pH	0.0063
TDS (ppm)	0–569	<500	±2% F.S.	0.0298
Turbidity (NTU)	0.00–7.00	<5.0	±5%	—
Temp (°C)	26.0–35.1	N/A	±0.5 °C	—

‘—’ denotes not applicable: no forecasting/RMSE evaluation was performed for turbidity or temperature, as the AI-driven trend-forecasting module (Section 4) targets pH and TDS only (Section 5).

**Table 4:** Comparative analysis with related works.

Feature	Ajakwe et al. (2023) [2]	Adeleke et al. (2023) [11]	Proposed HydroSense AI
Main focus	Connected-intelligence water monitoring	Embedded IoT-based water monitoring with ML	Hybrid IoT monitoring with forecasting and automation
Communication	IoT-connected architecture	Embedded IoT communication	Hybrid LoRa–Wi-Fi
Long-range capability	Not emphasised	Not emphasised	Yes
Power management	Limited	Limited	RTC-based duty cycling
Predictive analytics	No	Yes, ML-based	Yes, LSTM-based
Alerting	Monitoring-oriented	Data-driven outputs	Telegram + AI assistant
Closed-loop control	No	No	Yes
Deployment scope	Smart monitoring framework	Embedded monitoring system	Institutional-scale field deployment

## Data Availability

The raw telemetry datasets (pH, TDS, turbidity, and temperature) generated and analysed during the field deployment at the SXCCE campus, together with the firmware and the trained LSTM forecasting model, are available from the corresponding author on reasonable request. The processed data underlying the figures and tables in this article are included within the article.

## Abbreviations

The following abbreviations are used in this manuscript:

IoT	Internet of Things
LPWAN	Low-Power Wide-Area Network
LoRa	Long Range
RTC	Real-Time Clock
LSTM	Long Short-Term Memory
TDS	Total Dissolved Solids
NTU	Nephelometric Turbidity Units
RMSE	Root Mean Square Error
RSSI	Received Signal Strength Indicator
SNR	Signal-to-Noise Ratio
TinyML	Tiny Machine Learning
WHO	World Health Organization
